# Quality Assessment of Day-Old Chickens on the Broiler Farms of Hong Kong

**DOI:** 10.3390/ani12121520

**Published:** 2022-06-10

**Authors:** Omid Nekouei, Denis Yau, Brett MacKinnon, Ioannis Magouras, Anne Conan, Ibrahim Elsohaby, Surya Paudel, Dirk U. Pfeiffer

**Affiliations:** 1 Department of Infectious Diseases and Public Health, Jockey Club College of Veterinary Medicine and Life Sciences, City University of Hong Kong, Hong Kong SAR, China; brett.mackinnon@cityu.edu.hk (B.M.); ioannis.magouras@cityu.edu.hk (I.M.); ielsohab@cityu.edu.hk (I.E.); spaudel@cityu.edu.hk (S.P.); dirk.pfeiffer@cityu.edu.hk (D.U.P.); 2 The Centre for Applied One Health Research and Policy Advice (OHRP), City University of Hong Kong, Hong Kong SAR, China; denisyau@cityu.edu.hk (D.Y.); ayconan@cityu.edu.hk (A.C.)

**Keywords:** day-old chick, broiler, visual assessment, maternally derived antibody, biosecurity

## Abstract

**Simple Summary:**

Assessing the quality and determining maternal antibodies against regional pathogens in day-old chickens is important to optimize the production performance and management of flocks. So far, such information in Hong Kong is lacking. The present study was conducted to assess abnormalities in appearance, navel, crop filling, dehydration, body weight, and length uniformity in day-old chicks. In addition, the maternally derived antibody levels against relevant infectious diseases were measured. This study was the first in Hong Kong to provide a baseline for the quality of day-old chicks, and results in evidence-based management advice tailored to the farms. We advise the establishment of a well-managed broiler breeder farm and hatchery, in order to improve the health and productivity of the local broiler chicken industry.

**Abstract:**

The present study aimed to investigate the quality of newly hatched broiler chicks delivered to Hong Kong (imported or local), and to develop tailored recommendations to improve their management. During 2019–2021, 70 batches (34 imported from mainland China and 36 local) of one/three day old chicks on 11 broiler farms were studied. From each batch, 23 or 24 chicks (1647 in total) were assessed for abnormalities in appearance, navel, crop filling, dehydration, body weight, and length uniformity. Chicks were sacrificed, and yolk sac residues in three day old chicks were measured. Maternally derived antibody levels against Newcastle disease virus (NDV), infectious bursal disease virus (IBDV), and avian reovirus (ARV) were measured in all chicks using an enzyme-linked immunosorbent assay (ELISA). The proportion of abnormal navel in most batches is high (median: 59%), ranging between 0 and 100%. The average length of chicks within batches ranges between 16.3 and 20.7 cm, and their average weights are 31–38.5 g upon delivery to the farms. On average, imported batches have a higher body weight and length than their local counterparts. The average yolk-free weight varies between 45 and 55 g, which is significantly lower in local batches (33–43 g). The mean antibody titers against NDV and ARV are higher in imported batches than in the local ones. In contrast, the mean antibody titer against IBDV is significantly lower in the imported batches. Concerning the overall lower quality of local chicks compared to the imported batches, establishing a well-managed broiler breeder farm and a hatchery in Hong Kong is highly recommended to enhance the health and productivity of the local broiler chicken industry.

## 1. Introduction

A comprehensive and regular assessment of day-old chicks (DOCs) is necessary to enhance hatchery and broiler production performances. Even slight imbalances in chick quality can substantially impact the performance and profitability of the broiler farms. Various methods and parameters are considered in the DOC quality assessment, including the visual evaluation of physical traits, and measuring the level and uniformity of maternal antibodies against specific infectious agents that are relevant in the local contexts [1,2].

Nearly all poultry farms in Hong Kong can be categorized as semi-intensive, and formal consultation with poultry experts and veterinarians is minimal. On the other hand, the local poultry industry has been slowly consolidating in response to challenges and opportunities, leading to a gradual decrease in the number of farms [3]. Currently, there are 29 poultry farms (28 active) in Hong Kong, amounting to an average daily production of 10 to 11 thousand live poultry [4]. All of the farms solely rear indigenous broiler chickens. On some of the farms, mixed-age groups of birds, including broiler breeders, are kept together, but in a different housing. The local poultry farms face many health and production challenges, and there has been resistance to adopting modern technologies and production methods among some farmers. There is still no structured data collection system in most farms, and essential performance parameters are not monitored on a regular basis, which is a major hindrance to sustainable agriculture development. In 2019, the Centre for Applied One Health Research and Policy Advice (OHRP) at City University of Hong Kong, supported by the government of the Hong Kong Special Administrative Region (HKSAR), established the first ambulatory veterinary service for local poultry producers in Hong Kong, aiming to improve the health, production, and sustainability of the local poultry industry. The first mission of this team was to establish trust and specify the poultry value chain via regular visits, benchmarking the production indices, and providing evidence-based health and management advice to the farmers.

Currently, the local broiler farms in Hong Kong are supplied with DOCs from three sources: (1) importation from four breeders in mainland China; (2) six self-breeding broiler farms with their private hatcheries in Hong Kong; and (3) one active registered hatchery in Hong Kong (seven broiler farmers have a contract with this hatchery to meet their DOC demands). To effectively improve the health, welfare, and productivity of local chickens, the source and quality of DOCs must be evaluated. There is no formal documentation of the field information regarding the quality of the chicks provided by these suppliers in order to compare performance and support evidence-based management decisions made by the local farmers. The objectives of this study were to investigate and establish a baseline for the quality of DOCs supplied to broiler farms in Hong Kong by source (imported or local), and develop tailored recommendations to improve the management of DOCs based on their source.

## 2. Materials and Methods

### 2.1. Sampling and Laboratory Testing

Between May 2019 and March 2021, 50 batches of one day old chicks and 20 batches of three day old chicks (70 batches in total) supplied to 11 different farms (1–13 batches per farm) were surveyed, based on the access granted to the service team and other logistic considerations. Each batch consisted of the chicks purchased/delivered at a single time. The traditional definition of ‘DOC quality’ involves a combination of hatchability and three day mortality; however, the precise definition remains rather subjective. In our local context, chicks of either one or three days of age were considered DOC, and subjected to our quality assessments. Based on the formal consents obtained from the farmers and the animal ethics approval, we were granted the right to study a maximum of 24 chicks in each visit. Therefore, 23 or 24 chicks from each batch were selected and visually assessed for any abnormalities in appearance. Farmers were briefed and attempted to select DOCs representing all delivered boxes of chicks without any preliminary examinations (i.e., judgmental sampling). The chick length from the tip of the beak to the implantation of the third toe was measured, as described previously [5]. Navel quality was recorded as normal (clean and sealed), or abnormal. Crop filling was assessed through palpation and reported dichotomously. If the crop was full, soft, and round (yes); otherwise (no). Dehydration was reported (yes/no) by assessing the chickens’ feet to see whether their veins were protruding/dry or not [6]. After visual assessments, the chicks were sacrificed by cervical dislocation, and blood samples were taken via heart puncture. The sera were extracted from the blood samples and stored in a freezer at −20 °C. Within a week of sample collection, the frozen sera were sent to the Veterinary Diagnostic Laboratory at City University of Hong Kong, for the quantification of maternally derived antibody (MDA) levels against Newcastle disease virus (NDV), infectious bursal disease virus (IBDV), and avian reovirus (ARV), using commercially available enzyme-linked immunosorbent assay (ELISA) test kits (ID Screen^®^ 310 rue Louis Pasteur, 34790 Grabels, France), according to the manufacturer’s instructions. To determine yolk-free body weight in 3 day old chicks (72 h is the expected time for the yolk to be completely absorbed), residual yolks were removed, weighed, and subtracted from the total body weight.

### 2.2. Statistical Analysis

All data management and statistical analyses were performed using Stata v17 (StataCorp LLC, College Station, TX, USA). As several DOC suppliers have small shares in the local broiler market, we focused our study on batches from two major suppliers with the largest shares, to allow for meaningful comparisons of the quality parameters between the local and imported batches. One supplier imported chicks from mainland China, holding >80% of the market share in Hong Kong, and the other supplied DOCs from a local hatchery with seven contracted broiler farms. Frequency distributions of all quality parameters were evaluated by source (imported or local) using relevant tables and box plots.

All quantitative parameters were averaged at the batch level, and their batch-level means were calculated. The distributions of the mean values were assessed and compared between the two sources of DOCs (local vs. imported) using the two-sample *t*-test: length (cm), body weight (g), yolk-sac weight (g), yolk-free/net body weight (g), coefficient of variation of length (representing uniformity), and ELISA antibody titers for NDV, IBDV, and ARV.

## 3. Results

In total, 34 imported (23 one day old and 11 three day old) and 36 locally sourced (27 one day old and 9 three day old) batches were studied. The frequency distribution of qualitative parameters for the 1647 studied DOCs by the source is presented in Table 1. Crops were filled in nearly half of the three-day old batches from both sources (n = 9). The proportion of abnormal navel in most batches from the two sources is high, ranging between 0 and 100%. The proportion of dehydration (dry feet) in chicks from both sources is very low, indicating appropriate hydration of most DOCs in the study (Table 1).

**Figure 1:**
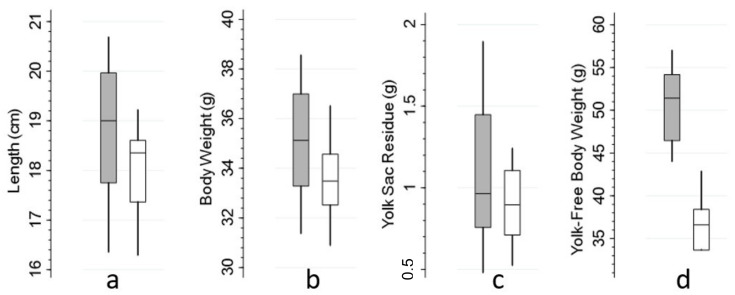
Distribution of the means of quality parameters at the batch level by source (imported in gray and local in white). Each box represents the interquartile range, including the median line. Whiskers represent the lowest and highest adjacent values (the most extreme values within 1.5 interquartile range of the nearer quartile).

**Figure 2:**
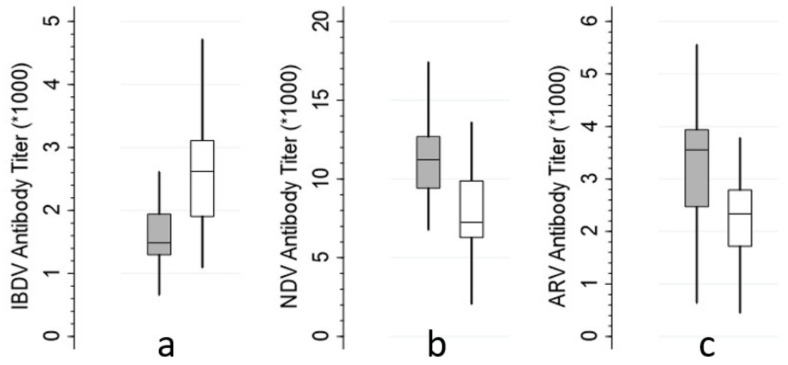
Distribution of the means of ELISA antibody titers for IBDV, NDV, and ARV at the batch level by source (imported in gray and local in white). Each box represents the interquartile range, including the median line. Whiskers represent the lowest and highest adjacent values (the most extreme values within 1.5 interquartile range of the nearer quartile). “*” = multiplied by.

The frequency distributions of the batch-level quantitative parameters and ELISA titers by source are illustrated in Figure 1 and Figure 2, respectively. Summary statistics (mean and SD) and statistical comparisons of these parameters by source (imported vs. local) are presented in Table 2.

The average length of DOCs within batches range between 16.3 and 20.7 cm, and their average weights varies from 31 to 38.5 g upon delivery to the farms (Figure 1a and Table 2). On average, body weights and lengths of the imported batches are higher than their local counterparts upon delivery (Table 2). In addition, imported batches have a lower variation of length (coefficient of variation) on average, indicating significantly higher uniformity in size (Table 2; *p* = 0.025). The average weight of yolk sac residue varies between 0.5 and 1.9 g in all batches of three day old chicks (Figure 1b). Most three day old batches (n = 17) have average yolk sac residues of <1.5 g. Although local batches are more uniform with respect to the absorption of yolk sac, compared to the imported batches, the difference in the residual weights is not significant (*p* = 0.265) (Figure 1c and Table 2). The average yolk-free/net body weight at day 3 in the majority of imported batches varies between 45 and 55 g, whereas it is significantly lower for local batches, 33–43 g (*p* < 0.001) (Figure 1d and Table 2).

The mean antibody titers for NDV and ARV are significantly higher in imported batches compared to the local batches (Table 2). However, the dispersions (uniformity) of the antibody titers are comparable between the two sources (Figure 2b,c). The majority of batch-level ELISA titers for IBDV from both sources (n = 51) are below 3000, the recommended threshold to reduce the burden of intensive vaccination in the early days of production by extending the passive immunity against IBDV field challenges (Figure 2a). The mean value of the batch-level IBDV titers is significantly lower in the imported batches compared to the local batches (*p* < 0.001) (Table 2). In addition, the coefficient of variation of the average titers for all batches is greater than 20, indicating a clear lack of uniformity in the maternal antibodies.

## 4. Discussion

The assessment of broiler chick quality is generally quite subjective. High-quality chicks are expected to be well-hydrated, active, and have a low mortality rate in the first five days of life (<0.5%). In addition, chicks should have uniform size and growth, adequate levels of maternal antibodies to the pathogens relevant in the region, and well-healed navels [7]. Our survey included several qualitative and quantitative parameters commonly used for scoring chick quality in hatchery management [5].

The broiler sector in Hong Kong produces a variety of ‘three-yellow chicken’ (sanhuangji) hybrid breeds that consumers value for their specific flavor and texture of the meat. These breeds have been developed since the 1970s, and consist of local shiqi roosters crossed with foreign female chickens over the course of several generations [8]. The breeds of ‘three-yellow chicken’ produced in Hong Kong are known as slow to intermediate growers, typically taking 90–120 days to reach their desired market weight. The local people in Hong Kong often prefer these breeds because they believe that the texture and flavor of the meat are improved due to the prolonged time to slaughter [9]. The imported DOCs from mainland China are genetically different compared to the local breeds. In general, the imported breeds are considered to be relatively fast growers, taking 60–70 days to reach their desired market size. On a global scale, most commercial poultry producers strive for a high growth rate and low feed conversion ratio on farms. This is the case with the imported broiler chicks from mainland China, where fast-growing chickens are selectively bred for higher productivity. Therefore, the observed differences in body weight, length, and net weight between local and imported batches can mainly be attributed to their distinct genetic characteristics. Overall, local DOC batches are significantly smaller in size (mean body weight and length) and less uniform than the imported batches. However, many farmers in Hong Kong prefer to raise slow-growing birds for their superior quality of meat and potential animal welfare benefits [10]. Since market-sized birds are typically sold for similar prices within Hong Kong, regardless of their source, rearing the faster-growing imported broiler chicks could have a substantial economic advantage.

High-quality chicks should have a clean and closed navel, with no signs of swelling or infection [7]. Poor navel quality can be due to suboptimal incubation conditions, and lead to increased levels of infections, mortality, and reduced growth rate [11]. Among others, infection of chicks with *Escherichia coli* leading to omphalitis is a very common problem in young broiler chicks [12]. Such conditions are often associated with an unhygienic environment [13]. Environmental factors related to sub-optimal management practices can impact the growth and overall health of the hatchlings. Other risk factors for poor navel quality include inadequate regulation of incubation temperature or humidity. The high proportion of abnormal navels in our study suggests the need for a thorough review of the current hatchery management and brooding practices, in order to make pragmatic improvements. The low proportion of crop filling also indicates the possibility of poor brooding practices on the farms. Overall, this highlights the need for close ties between poultry farmers and local veterinarians to get advice on appropriate housing and husbandry practices.

Imported DOCs have a higher yolk-free body weight than locally supplied chicks, and there are no substantial differences in the residual yolk sac weight between the two sources. Although these parameters are commonly measured to assess chick quality in the poultry sector, their usefulness for predicting future growth is unclear [5]. Powell and Bowman [14] report a positive correlation between the weight of DOCs and slaughter weight, which is not supported by more recent studies [15,16,17]. However, a positive association is found between the weight of 7 to 10 day old chicks and their weight at day 42 [16]. Many studies document a positive association between chick length and body weight at 42 days of age [17,18,19,20]. A positive correlation is also reported between chick length and yolk-free body weight [5,21], likely due to higher yolk sac utilization efficiency [22].

Starting at three days of age and over the course of 180 days, most local broiler breeder chickens in Hong Kong are vaccinated against several important viral pathogens, including ARV, highly pathogenic avian influenza viruses (H5 and H7), infectious bronchitis virus (IBV), IBDV, low pathogenic avian influenza viruses (H9), Marek’s disease virus (MDV), and NDV. High and uniform levels of antibodies against these pathogens in broiler chicks determine the levels of protection against these common pathogens, and reduce adverse vaccine reactions [23]. The MDAs may last up to two weeks after hatching [24]. Low levels and/or lack of uniformity in MDAs among DOCs may result in early susceptibility to various infections. In mainland China, broiler breeders are commonly vaccinated at around a week of age with live attenuated NDV and ARV vaccines, and receive boosters, at appropriate intervals, with killed or live attenuated vaccines until 270 days of age (see Tables S1–S3 for details). This vaccination strategy increases the level of MDA in the broiler chicks from vaccinated breeders. However, in Hong Kong, breeders often are not vaccinated beyond 180 days, and vaccination is less frequent. This may explain why locally sourced batches of DOCs have lower titers for NDV and ARV than imported chicks. In contrast, the local batches in our study have higher MDA titers of IBDV compared to the imported batches. In the present study, local broiler breeders are primed with modified-live vaccines against IBDV, and receive a booster with a killed vaccine at 180 days of age. In mainland China, however, the broiler breeders were not primed with a live attenuated vaccine because IBD was not a major concern to the producers at the time. The local broiler breeder farms involved in this study had approximately 15 batches of breeder chickens in production, including multiple age groups, and each batch had an average of 300 hens. Hence, the breeder farms collected their hatching eggs from different age groups of hens, which leads to large fluctuations (low uniformity) in size and the MDA titers of the DOC batches delivered to the broiler farms. Therefore, establishing an independent broiler breeder farm to supply high-quality uniform DOCs to the broiler industry in Hong Kong could be very beneficial. It is also recommended that all broiler breeder farms provide the vaccination program of their breeder flocks and breeders’ ages for the imported DOCs to the local veterinarians, in order to enable a more meaningful interpretation of the quality and maternal immunity status of the imported batches. This could further support more effective, evidence-based management decisions, and enhance the overall performance of the local broiler farms.

Although there is no universal vaccination strategy for broiler breeders, it is generally accepted to prime the broiler breeders with live attenuated vaccines, followed by boosters with inactivated vaccines to achieve high and uniform MDA for their progeny [24]. Our ELISA titers for IBDV and ARV are quite variable, and lower than the recommended protection thresholds for most batches, regardless of their source. There are many different vaccine serotypes for ARV with varied cross-protection, and the commercially available ELISA test used in our study could not differentiate between these differences. In Hong Kong, we recommend using a ‘strain 1133 live ARV vaccine’ for priming the breeders, and boosting their immunity twice with bivalent (strains 1733 and 2408) inactivated ARV vaccine, six to eight weeks before egg production.

IBDV is one of the most important and prevalent immunosuppressive pathogens in poultry production worldwide. Effective control of IBD demands providing passive and active immunity to susceptible chickens, and maintaining all relevant biosecurity measures to reduce the field challenges. It is shown that the optimal timing of vaccine administration in broiler chickens is critical, and depends on the levels of MDA [25]. The high variation of MDA levels in the chicks suggests the need for vaccinating each broiler flock twice or more to induce homogenous protection in all birds. Usually, Hong Kong farmers follow a standard vaccination program that is not tailored to the specific conditions of their farms and the immunity status of the birds. Poultry veterinarians in Hong Kong should assist farmers with creating an effective vaccination strategy against IBDV by regularly monitoring the levels of maternal antibodies in DOCs.

The levels of MDA against NDV in the imported DOCs are significantly higher than the local batches, which can mainly be attributed to the intensive NDV vaccination programs in mainland China. As Newcastle disease is endemic in the region [26], most broiler breeder farms in mainland China apply intensive vaccination against NDV using live vaccines, even during the production stage. Vaccination against NDV cannot prevent the infection; however, it can limit the severity of the disease [24]. Obtaining hatching eggs from multiple producers, who pursue different vaccination programs, with varying ages of the broiler breeders, could further decrease the uniformity of MDA levels in a batch. Inactivated multivalent vaccines (including NDV, IBV, IBDV, and ARV) can be considered at off-peak production in broiler breeders to level-up the MDA of the DOCs from multiple breeders.

## 5. Conclusions

Regular monitoring and assessing of the health and quality parameters of DOCs on local broiler farms are highly recommended to provide evidence-based advice tailored to the field condition. With respect to the overall lower quality of local DOCs compared to the imported batches, there is a need for well-managed local broiler breeder farms and a more advanced hatchery in Hong Kong, in order to enhance the health and productivity of the local chicken farms. The baseline information provided in this study is useful for further research in the field. The practical recommendations from this study may be applied to similar production systems in mainland China, and other countries in southeast Asia.

## Figures and Tables

**Table 1:** Frequency distribution of day-old chicks (n = 1647) by the categorical quality parameters in the study.

Parameter	Age (Day) ^1^	Level	Source		Total
			Imported	Local	
Navel quality	1				976
		Abnormal	141	310	
		Normal	290	235	
	3				448
		Abnormal	81	112	
		Normal	183	72	
Dehydrated	1				831
		No	330	378	
		Yes	29	94	
	3				423
		No	240	125	
		Yes	0	58	
Crop-filled	3				444
		No	39	96	
		Yes	221	88	

^1^ Chicks were assessed from two age groups of one and three (all categorized as day-old chicks).

**Table 2:** Summary statistics and comparison of the batch-level quality parameters by source (imported = I vs. local = L) for the 70 batches of day-old chicks in the study.

Variable	Age (Day) ^1^	Source	Number of Batches	Mean	SD	*p*-Value ^2^
Length (cm)	1	I	20	18.9	1.3	0.017
		L	25	18.1	0.8	
Body weight (g)	1	I	20	35.0	2.2	0.016
		L	25	33.6	1.6	
Yolk sac residual (g)	3	I	11	1.1	0.5	0.265
		L	8	0.9	0.3	
Net weight (g)	3	I	11	49.6	7.1	<0.001
		L	8	35.6	5.4	
CV of length (%)	1	I	20	3.2	0.18	0.025
		L	25	3.9	0.21	
IBDV titer	1 & 3	I	33	1756.9	848.4	<0.001
		L	36	2680.1	891.3	
NDV titer	1 & 3	I	33	11,255.6	3278.8	<0.001
		L	36	7664.3	2831.9	
ARV titer	1 & 3	I	33	3781.2	2121.1	0.001
		L	35	2341.5	1023.7	

^1^ Chicks from 1 and 3 day old batches are included depending on the quality parameter of interest. ^2^ *p*-values result from two sample *t*-tests, comparing the means of each variable between the two sources of chicks (imported vs. local); *p*-value < 0.05 is considered statistically significant.

## Data Availability

The data will be made available upon request after acceptance of the manuscript via the journal’s server.

## Supplementary Materials

The following supporting information can be downloaded at: https://www.mdpi.com/article/10.3390/ani12121520/s1, Table S1: Current vaccination program for avian reovirus (ARV), highly pathogenic avian influenza (HPAI), infectious bronchitis (IB), infectious bursal disease (IBD/G), Marek’s disease (MD), Newcastle disease (ND), and avian metapneumovirus (AmPV) in broiler breeders in Hong Kong; Table S2: Current vaccination program for avian reovirus (ARV), infectious bronchitis (IB), infectious bursal disease (IBD/G), and Newcastle disease (ND) in broiler breeders in Mainland China. Information provided in this table is based on the farmers’ best knowledge regarding the suppliers; Table S3: Suggested vaccination program for avian reovirus (ARV), highly pathogenic avian influenza (HPAI), infectious bronchitis (IB), infectious bursal disease (IBD/G), Marek’s disease (MD), Newcastle disease (ND), chicken anemia virus (CAV), and avian metapneumovirus (AmPV) in the only broiler breeder farm in Hong Kong.
