# Supplementary material for: Quality Assessment of Day-Old Chickens on the Broiler Farms of Hong Kong
Source: Animals (Basel). 2022 Jun 10;12(12):1520. doi: 10.3390/ani12121520 (PMC9219531; doi:10.3390/ani12121520)
Supplement: Supplementary file 1 [file animals-12-01520-s001.zip › animals-1760566-supplementary.pdf]

Supplementary materials

Table S1. Current vaccination program for avian reovirus (ARV), highly pathogenic avian influenza (HPAI), infectious bronchitis (IB), infectious bursal disease (IBD/G), Marek's disease (MD), Newcastle disease (ND), and avian metapneumovirus (AmPV) in broiler breeders in Hong Kong.

**Age (day)**	**Vaccine**	**Type**[1]	**Strain**	**Dosage**	**Route**[2]
3	MD<br>IBD+ND	Cell-associated (CA)/herpes virus vector	CVI988<br>rHVT-ND-IBD	1:1<br>0.2ml	SC (hatchery)
	ND+IB	Live	Ma5+Clone30	1 dose	IN
7	ND+IBD	Live	LaSota+Vac-ST	1:1 0.5 ml	SC
14	HPAI	Killed	H5+H7	0.5 ml	SC
17	ND+LPAI	Killed	LaSota+H9	0.5 ml	SC
22	ILT	Live	CEO	1 dose	IO
70	AmPV	Live	Rhino-CV	1 dose	IO
140	AmPV	Killed	BUT1#8544	0.5 ml	IM
140	ND+LPAI	Killed	LaSota+H9	0.5 ml	IM
180	HPAI	Killed	H5+H7	0.5 ml	IM
180	ND+IB+G+Reo	Killed	Clone30+M41+D78+1733+2408	0.5 ml	IM

[1]Live = live attenuated vaccine; killed = inactivated vaccine
[2]Route of administration = subcutaneous (SC); intranasal (IN); intraocular (IO); and intramuscular (IM)

Table S2. Current vaccination program for avian reovirus (ARV), infectious bronchitis (IB), infectious bursal disease (IBD/G), and Newcastle disease (ND) in broiler breeders in mainland China. Information provided in this table is based on the farmers' best knowledge regarding the suppliers.

Age (day)	Vaccine	Type[1]	Strain	Dosage	Route[2]
1	ND+IB	Live	LaSota+QX		Spray
1	ND+IBD+H9	Killed	LaSota+B87+H9	0.15ml	SC
5	Reo	Live	1133	0.2ml	SC
10	ND+IB	Live	LaSota+QX	1dose	IO
10	ND+IB+IBD+H9	Killed	NDg7+QX+B87+H9?	0.3ml	SC
40	ND	Live	LaSota	2doses	IO
50	ND+H9+Adeno	Killed	NDg7+H9+Adeno(S?)	0.5ml	IM
60	Reo	Live	1133	0.2ml	SC
125	ND+H9+Adeno	Killed	NDg7+H9+Adeno(S?)	0.5ml	IM
140	ND	Live	LaSota	3doses	IO
155	ND+IB+IBD+Reo	Killed	LaSota+QX+VP2+1133?	0,5ml	IM
165	ND+IB	Live	LaSota+QX	1dose	IO
240	ND+IB	Live	LaSota+QX	1dose	IO
270	ND+IB	Live	LaSota+QX	1dose	IO

[1]Live = live attenuated vaccine; killed = inactivated vaccine

[2]Route of administration = subcutaneous (SC); intranasal (IN); intraocular (IO); and intramuscular (IM)

Table S3. Suggested vaccination program for avian reovirus (ARV), highly pathogenic avian influenza (HPAI), infectious bronchitis (IB), infectious bursal disease (IBD/G), Marek's disease (MD), Newcastle disease (ND), chicken anemia virus (CAV), and avian metapneumovirus (AmPV) in the only broiler breeder farm in Hong Kong.

Age (day)	Vaccine	Type[1]	Strain	Dosage	Route[2]
1	MD	CA / HVT vector	CVI988 +rHVT-ND-IBD	1:1 0.2ml	SC (hatchery)
1	ND+IB	Live	Ma5+Clone30	1 dose	IN/Spray in hatchery
1	AmPV	Live	Rhino CV	1 dose	IN/Spray in hatchery
7	ND+IBD	Live	LaSota+Vac-ST	1:1 0.5 ml	SC
10	HPAI	Killed	H5+H7	0.5 ml	SC
14	IB	Live	IB 4/91	1 dose	IN
17	ND+LPAI	Killed	LaSota+H9	0.5 ml	SC
22	ILT	Live	CEO	1 dose	IO
70	CAV	Live	CAV P4	0.2 ml	IM
90	ND+IB+G+Reo	Killed	Clone30+M41+D78+1733+2408	0.5 ml	IM
120	AmPV	Killed	BUT1#8544	0.5 ml	IM
140	ND+LPAI	Killed	LaSota+H9	0.5 ml	IM
160	HPAI	Killed	H5+H7	0.5 ml	IM
180	ND+IB+G+Reo	Killed	Clone30+M41+D78+1733+2408	0.5 ml	IM

[1]Live = live attenuated vaccine; killed = inactivated vaccine
[2]Route of administration = subcutaneous (SC); intranasal (IN); intraocular (IO); and intramuscular (IM)
